# Supplementary material for: New internationalization paths of Chinese brands: A configurational study
Source: PLoS One. 2023 Sep 29;18(9):e0292253. doi: 10.1371/journal.pone.0292253 (PMC10540969; doi:10.1371/journal.pone.0292253)
Supplement: Attachment — Submitted filename: Plos One comments.docx [file pone.0292253.s002.docx]

This manuscript focus on the internationalization of Chinese brands. This topic is worthy to study. The authors have made some contributions to Chinese areas.

Here are some suggestions to the authors.

The title should be direct to reveal the internationalization of Chinese brands. The whole paper should clearly pay attention to this research object too. As second largest economy, Chinese brands have enough potential to be studied. The authors can write some words about implication for emerging economy in the end.

**Introduction**

The authors should integrate more literature about internationalization Chinese companies. “Research gap” in the part “Introduction” should be in the last paragraph of “Introduction”. There are too many theoretical perspective in “Introduction” and in the whole paper. There are too many research questions. The authors may highlight 2 or three questions.

**Theoretical Basis**

As above, there two many theories, for example, brand equity, brand identity, spring board, born global, RBV. “Cross the Chasm” has a special context. There are many literatures about “Liability of origin, liability of foreignness”. The authors may select one or two theories, and conduct literature review in detail, find research gap.

**Theoretical Analysis**

Table 6.

The brands in Table 6 come from different industries, the companies have different histories. For example, in the beginning Huawei supplied only telecommnication equipment, namely B to B. Hisense mainly B to C. Some brands are platform. Xiaomi has completely different business model. Therefore, the authors should be aware of these differences as the authors discuss about paths of brand internationaliztions.

**5.2 Research Contribution**

The authors may conduct literature review, find research gap, the rewrite theoretical contributions.

Reference

The literature in the reference should have unified format.
