# Supplementary material for: New internationalization paths of Chinese brands: A configurational study
Source: PLoS One. 2023 Sep 29;18(9):e0292253. doi: 10.1371/journal.pone.0292253 (PMC10540969; doi:10.1371/journal.pone.0292253)
Supplement: Attachment — Submitted filename: Response Letter.docx [file pone.0292253.s003.docx]

Dear Editors and Reviewers,

Thank you for your letter and for the reviewers' comments concerning our manuscript entitled "How to Travel on the Road of Internationalization of Chinese Brands" with Manuscript Number: PONE-D-22-33203. Those comments are all valuable and very helpful for revising and improving our paper, as well as the important guiding significance to our research. We have studied the comments carefully and have made corrections which we hope meet with approval. The revised portions are highlighted in grey in the paper. The main corrections in the paper and the responses to the reviewers' comments are as follows.

**1. Response to comment:** ("The title should be direct to reveal the internationalization of Chinese brands. The whole paper should clearly pay attention to this research object too. As second largest economy, Chinese brands have enough potential to be studied. The authors can write some words about implication for emerging economy in the end.")

**Response:** Thanks to the reviewers for pointing out this problem. Our previous perspective is indeed too large compared with our research samples that are all Chinese brands. We fully accept the suggestions. We have made some adjustments as follow.

- ("The title should be direct to reveal the internationalization of Chinese brands. "): We have modified the title to: "How to Travel on the Road of Internationalization of Chinese Brands" (lines 1)，
- ("The whole paper should clearly pay attention to this research object too."): we have added more literatures on the internationalization of Chinese brands in the Introduction section (lines 21-86). Relevant details have also been adjusted (lines 11, 17, 18, etc.).
- ("The authors can write some words about implication for emerging economy in the end."): we have written some words about implication for emerging economies in subsection 5.3. Practical Enlightenment (lines 572-574).

The relevant modifications are in section 1. Introduction and subsection 5.3. of the manuscript.

**2. Response to comment:** ("The authors should integrate more literature about internationalization Chinese companies. “Research gap” in the part “Introduction” should be in the last paragraph of “Introduction”. There are too many theoretical perspectives in “Introduction” and in the whole paper. There are too many research questions. The authors may highlight 2 or three questions.")

**Response:** Thanks to the reviewers for pointing out this problem. Our previous literature integration is indeed fragmented. According to the reviewers' suggestions, we have focused on the internationalization of Chinese brands and adjusted the research gap to the end of the Introduction section, and reduced the number of research questions from 4 to 2 to make our research more targeted.

- ("The authors should integrate more literature about internationalization Chinese companies."): we have organized the literature on brand internationalization of Chinese enterprises form three perspectives: “go global”, “be local” and “lead global” (lines 31-81).
- ("“Research gap” in the part “Introduction” should be in the last paragraph of “Introduction”."): the research gap has been adjusted to the end of “Introduction” (lines 81-86).
- ("There are too many theoretical perspectives in “Introduction” and in the whole paper."): we have focused on the internationalization path of Chinese brands (lines 68-97), and our research theory reduced to two theories: brand empowerment and boundary spanning (lines 98-142).
- ("There are too many research questions. The authors may highlight 2 or three questions."): our research questions have also been refined to: (1) What are the key drivers of Chinese brands to achieve brand internationalization; (2) What are the paths for the Chinese brand internationalization (lines 92-94).

The relevant modifications are in section 1. Introduction and section 2. Research Model of the manuscript.

**3. Response to comment:** ("As above, there too many theories, for example, brand equity, brand identity, spring board, born global, RBV. “Cross the Chasm” has a special context. There are many literatures about “Liability of origin, liability of foreignness”. The authors may select one or two theories, and conduct literature review in detail, find research gap.")

**Response:** Thank you for pointing out that our theoretical perspective is quite scattered and the structure is not clear enough. We have integrated the literature review into the Introduction section to make the structure of the article clearer and more compact.

- ("As above, there too many theories, for example, brand equity, brand identity, spring board, born global, RBV."): There are indeed too many theories in this article, so we have narrowed them down and introduced the brand empowerment theory and the boundary spanning theory in detail (lines 98-142).
- ("“Cross the Chasm” has a special context."): We are very sorry for our negligence. Cross the Chasm has a special context that is applicable to consumers' acceptance of new products, not brand internationalization. We have modified our main theory to boundary spanning theory (lines 98-142).
- ("The authors may select one or two theories, and conduct literature review in detail, find research gap."): We have conducted the literature review as suggested and found research gap: “This means that there is a certain research gap in the multi-stage brand internationalization path research based on quantitative data and considering the interactive effect of multiple drivers”. Due to the theoretical support required for the proposed model of QCA research is very strict, we have presented a detailed explanation of the proposed research model as a chapter to reflect the exact of our model (section 2. Research Model, lines 98-167), and incorporated the literature review section into the Introduction section (lines 35-86). It is hoped that editors and reviewers can allow us to make such adjustments.

The relevant modifications are in section 1. Introduction and section 2. Research Model of the manuscript.

**4. Response to comment: (**"Table 6: The brands in Table 6 come from different industries, the companies have different histories. For example, in the beginning Huawei supplied only telecommunication equipment, namely B to B. Hisense mainly B to C. Some brands are platform. Xiaomi has completely different business model. Therefore, the authors should be aware of these differences as the authors discuss about paths of brand internationalization.")

**Response:** Thanks to the reviewers for pointing out this problem. We do not consider that readers' different understanding of the enterprise that makes our result confusing. Therefore, based on the suggestions of reviewers, we have added 4.4.1. subsection to provide a detailed explanation of the background of the cases.

- ("The brands in Table 6 come from different industries, the companies have different histories "): The results shown in Table 7 (Table 6 of the original manuscript) are a little confusing. In order to provide readers with a clear understanding of the background of cases covered by path in the output result of QCA, we have added section 4.4.1. Analysis of cases covered by path (lines 303-351). Based on the theoretical framework of this article, we have provided a background introduction to the cases covered by each path (lines 303-351).
- ("in the beginning Huawei supplied only telecommunications equipment, named B to B. "): we have introduced it in section 4.5. that Huawei's development process after starting B to C is in line with the L-M-Co path (lines 425-437).
- In order to demonstrate the reason for the merging of Path 1 and Path 2, we have added two minimal formulas to reflect the similarity of the two paths by extracting common factors from the formulas for Path 1 and Path 2 (lines 260-263, 353-358).

The relevant modifications are in subsection 4.2, 4.4 and 4.5 of the manuscript.

**5. Response to comment:** ("5.2 Research Contribution: The authors may conduct literature review, find research gap, then rewrite theoretical contributions.")

**Response:** Thanks to the reviewers for pointing out this problem. We did not integrate the literature review, research gap, and theoretical contributions well before. Therefore, we have conducted literature review (lines 35-87), found research gap (lines 82-87). Based on the research gap, we have rewritten theoretical contributions (lines 542-572).

The relevant modifications are in subsection 5.2. Theoretical Contributions of the manuscript.

**6. Response to comment:** ("Reference: The literature in the reference should have unified format.")

**Response:** Thanks to the reviewers for pointing out this problem. We have carefully examined each literature and found that there is indeed missing page section in some literatures. We have corrected the malformed citation (lines 602-743).

The relevant modifications are in section References of the manuscript.

We have tried our best to improve the manuscript and made some changes. The revised portions are highlighted in grey in the paper.

We appreciate the Editors' warm work and the Reviewers' valuable comments earnestly and hope that the correction will meet with approval.

Once again, thank you very much for your comments and suggestions.
